# Increased risks for mental disorders among LGB individuals: cross-national evidence from the World Mental Health Surveys

**DOI:** 10.1007/s00127-022-02320-z

**Published:** 2022-07-19

**Authors:** Jan-Ole H. Gmelin, Ymkje Anna De Vries, Laura Baams, Sergio Aguilar-Gaxiola, Jordi Alonso, Guilherme Borges, Brendan Bunting, Graca Cardoso, Silvia Florescu, Oye Gureje, Elie G. Karam, Norito Kawakami, Sing Lee, Zeina Mneimneh, Fernando Navarro-Mateu, José Posada-Villa, Charlene Rapsey, Tim Slade, Juan Carlos Stagnaro, Yolanda Torres, Ronald C. Kessler, Peter de Jonge, The WHO World Mental Health Survey collaborators, Sergio Aguilar-Gaxiola, Ali Al-Hamzawi, Jordi Alonso, Laura Helena Andrade, Lukoye Atwoli, Corina Benjet, Guilherme Borges, Evelyn J. Bromet, Ronny Bruffaerts, Brendan Bunting, Jose Miguel Caldas-de-Almeida, Graça Cardoso, Somnath Chatterji, Alfredo H. Cia, Louisa Degenhardt, Koen Demyttenaere, Silvia Florescu, Giovanni de Girolamo, Oye Gureje, Josep Maria Haro, Meredith Harris, Hristo Hinkov, Chi-yi Hu, Peter de Jonge, Aimee Nasser Karam, Elie G. Karam, Norito Kawakami, Ronald C. Kessler, Andrzej Kiejna, Viviane Kovess-Masfety, Sing Lee, Jean-Pierre Lepine, John McGrath, Maria Elena Medina-Mora, Zeina Mneimneh, Jacek Moskalewicz, Fernando Navarro-Mateu, Marina Piazza, Jose Posada-Villa, Kate M. Scott, Tim Slade, Juan Carlos Stagnaro, Dan J. Stein, Margreet ten Have, Yolanda Torres, Maria Carmen Viana, Daniel V. Vigo, Harvey Whiteford, David R. Williams, Bogdan Wojtyniak

**Affiliations:** 1 Department of Developmental Psychology, University of Groningen, Grote Kruisstraat 2/1, 9712 TS Groningen, The Netherlands; 2 Interdisciplinary Center Psychopathology and Emotion Regulation, University Medical Center Groningen, University of Groningen, Groningen, The Netherlands; 3 Department of Pedagogy and Educational Sciences, University of Groningen, Groningen, The Netherlands; 4 Center for Reducing Health Disparities, UC Davis Health System, Sacramento, CA USA; 5 Health Services Research Unit, IMIM-Hospital del Mar Medical Research Institute, Barcelona, Spain; 6 Pompeu Fabra University (UPF), Barcelona, Spain; 7 CIBER en Epidemiología y Salud Pública (CIBERESP), Barcelona, Spain; 8 National Institute of Psychiatry Ramón de la Fuente Muñiz, Mexico City, Mexico; 9 School of Psychology, Ulster University, Londonderry, UK; 10 Lisbon Institute of Global Mental Health and Chronic Diseases Research Center, NOVA Medical School, NOVA University of Lisbon, Lisbon, Portugal; 11 National School of Public Health, Management and Development, Bucharest, Romania; 12 Department of Psychiatry, University College Hospital, Ibadan, Nigeria; 13 Department of Psychiatry and Clinical Psychology, Faculty of Medicine, Balamand University, Beirut, Lebanon; 14 Department of Psychiatry and Clinical Psychology, St George Hospital University Medical Center, Beirut, Lebanon; 15 Institute for Development Research Advocacy and Applied Care (IDRAAC), Beirut, Lebanon; 16 Department of Digital Mental Health, Graduate School of Medicine, The University of Tokyo, Tokyo, Japan; 17 Department of Psychiatry, Chinese University of Hong Kong, Tai Po, Hong Kong; 18 Survey Research Center, Institute for Social Research, University of Michigan, Ann Arbor, MI USA; 19 IDRAAC, Beirut, Lebanon; 20 UDIF-SM, Subdirección General de Planificación, Innovación y Cronicidad, Servicio Murciano de Salud, IMIB-Arrixaca, CIBERESP-Murcia, Murcia, Spain; 21 Colegio Mayor de Cundinamarca University, Faculty of Social Sciences, Bogota, Colombia; 22 Department of Psychological Medicine, University of Otago, Dunedin, Otago New Zealand; 23 The Matilda Centre for Research in Mental Health and Substance Use, University of Sydney, Sydney, Australia; 24 Departamento de Psiquiatría y Salud Mental, Facultad de Medicina, Universidad de Buenos Aires, Buenos Aires, Argentina; 25 Center for Excellence on Research in Mental Health, CES University, Medellin, Colombia; 26 Department of Health Care Policy, Harvard Medical School, Boston, MA USA; 27 Department of Developmental Psychology, University of Groningen, Groningen, The Netherlands

**Keywords:** Epidemiology, Health status disparities, Mental disorders, Cross-national, Sexual orientation

## Abstract

**Purpose:**

Lesbian, gay, and bisexual (LGB) individuals, and LB women specifically, have an increased risk for psychiatric morbidity, theorized to result from stigma-based discrimination. To date, no study has investigated the mental health disparities between LGB and heterosexual AQ1individuals in a large cross-national population-based comparison. The current study addresses this gap by examining differences between LGB and heterosexual participants in 13 cross-national surveys, and by exploring whether these disparities were associated with country-level LGBT acceptance. Since lower social support has been suggested as a mediator of sexual orientation-based differences in psychiatric morbidity, our secondary aim was to examine whether mental health disparities were partially explained by general social support from family and friends.

**Methods:**

Twelve-month prevalence of DSM-IV anxiety, mood, eating, disruptive behavior, and substance disorders was assessed with the WHO Composite International Diagnostic Interview in a general population sample across 13 countries as part of the World Mental Health Surveys. Participants were 46,889 adults (19,887 males; 807 LGB-identified).

**Results:**

Male and female LGB participants were more likely to report any 12-month disorder (OR 2.2, p < 0.001 and OR 2.7, p < 0.001, respectively) and most individual disorders than heterosexual participants. We found no evidence for an association between country-level LGBT acceptance and rates of psychiatric morbidity between LGB and heterosexualAQ2 participants. However, among LB women, the increased risk for mental disorders was partially explained by lower general openness with family, although most of the increased risk remained unexplained.

**Conclusion:**

These results provide cross-national evidence for an association between sexual minority status and psychiatric morbidity, and highlight that for women, but not men, this association was partially mediated by perceived openness with family. Future research into individual-level and cross-national sexual minority stressors is needed.

**Supplementary Information:**

The online version contains supplementary material available at 10.1007/s00127-022-02320-z.

## Introduction

Lesbian, gay and bisexual (LGB) individuals are at an increased risk for mental health issues as compared to heterosexual individuals [1–7]. For example, mood, anxiety, and substance use disorders are at least 1.5 times more likely to occur in LGB individuals than in heterosexual individuals [2, 3, 6, 8]. Among LGB individuals, gender differences in psychiatric morbidity have also been documented: compared to heterosexual participants, gay and bisexual (GB) men have been found to experience a higher risk for mood disorders [4, 5], while lesbian and bisexual (LB) women appear to be more adversely affected by substance-related issues [2, 3, 5]. Overall, the risk for LGB individuals to be diagnosed with at least one disorder in the last 12 months appears to be twice as high as compared to heterosexual individuals [5]. In addition, among sexual minorities, bisexual individuals have been found to have especially high risks for experiencing adverse mental health outcomes [9].

Notably, these mental health disparities between LGB and heterosexual individuals have been well documented by national studies [4, 5, 7, 8, 10–12], which have predominantly focused on Australian [10, 11], European [4, 5, 7], and North-American [8, 11] contexts. However, differences in socio-structural factors, such as the social acceptance of LGB individuals, have been found to be related to country-level effects on health [13, 14] and happiness [15] among sexual minority individuals. Similarly, there is evidence of cross-national [16, 17], and within-US state-level effects on psychiatric morbidity among sexual minority individuals [18, 19]. However, because of cross-national differences in the prevalence of mental disorders generally, it is unclear if cross-national differences in mental health outcomes among LGB individuals are actually specific to LGB individuals or simply reflect more general differences in the prevalence of mental disorders across countries. Some cross-national evidence for disparities in mental health outcomes (specifically depression, anxiety disorders, and alcohol use disorders) between LGB and heterosexual individuals also comes from meta-analyses [20, 21]. However, studies assessing mental health disparities between LGB and heterosexual individuals across a broad range of mental disorders in a cross-national sample, including Western and non-Western countries, are lacking [21].

### Minority stress and social support as a mediator

In addition to societal stressors related to their stigmatized sexual orientation, there is consistent evidence linking the elevated rates of psychiatric morbidity among LGB individuals to the experience of individual-level stressors (e.g. sexuality-based violence, as well as internalized societal stigma) [22–26]. In addition to having a cumulative effect on psychiatric morbidity [23, 27], it has been suggested that minority stress may result in elevated emotion dysregulation, interpersonal issues, and cognitive processes that ultimately increase LGB people’s risk for psychopathology [27]. According to Hatzenbuehler’s psychological mediation framework, sexual minority individuals may experience poorer social relationships as a consequence of social rejection and isolation due to their sexual minority status [28], which in return might reduce their resources to cope with general life stressors.

Indeed, compared to heterosexual individuals, sexual minorities have been found to report lower perceived social support in general [11, 29] and to report less social support from family as compared to friends [11]. In line with the psychological mediation framework, higher social support has been found to result in better coping and resilience among sexual minority individuals [29, 30]. Specifically, support from family as compared to friends appears to be an important predictor of the mental health of LGB individuals [31]. Conversely, there is some evidence that lower social support may mediate the adverse effects of minority stress on health generally [32], and mental health specifically [33, 34]. In addition, there are some findings that suggest that social support may mediate the relationship between sexual minority status and psychiatric morbidity among young men, but not women [28].

### Current study

The primary aim of the current study was to contribute to this literature by examining global mental health disparities between sexual minority and heterosexual individuals. The WHO World Mental Health Survey Initiative (WHMS) consists of a cross-national dataset that includes a comprehensive set of mental disorders. As such, it offers a unique opportunity to study disparities in mental health outcomes based on sexual orientation in the largest cross-national general population sample investigating the most comprehensive set of disorders to date. Based on previous national population studies, we expected LGB individuals to be more likely to report adverse mental health outcomes across multiple disorder groups as compared to heterosexual individuals. In addition, we explored the association between country-level social acceptance and the increased risk for mental disorders among LGB individuals compared to heterosexual individuals. The second aim of this study was to investigate the role of social support as a potential mediating factor for mental health issues among LGB individuals.

## Methods

### Sample

Data came from the WHO World Mental Health Surveys [35]. Translated versions of the WHO Composite International Diagnostic Interview (CIDI) version 3.0 [36] were administered in 29 surveys across the world through stratified multistage clustered area probability household sampling between 2001 and 2012 (average response rate 69.5%, range 45.9–97.2%) based on Census area data, with the exact recruitment and data collection procedures varying somewhat by country [37]. Adults from the non-institutionalized population were selected to generate population-representative samples (for an overview of the individual country sub-samples see Supplemental Table 1). A total of 13 surveys assessing sexual orientation were included in the current study. All surveys within the World Mental Health Survey Initiative are commonly presented by country income group; surveys were grouped into a low/middle-income country group (Colombia, Colombia (Medellin), Mexico, Peru, and Romania) and a high-income country group (Argentina, Australia, Japan, New Zealand, Northern Ireland, Portugal, Spain (Murcia), United States). Country income categories were based on the World Bank criteria at the time of each survey [38].

**Table1:** Prevalence of heterosexual, gay/bisexual, and lesbian/bisexual, and associated demographics

	Male		Female	
	Heterosexual	GB	Heterosexual	LB
	(*N* = 19,530)	(*N* = 357)	(*N* = 26,552)	(*N* = 450)
	% (SE)	% (SE)	% (SE)	% (SE)
Education status				
Low	14.4 (0.34)	11.1 (2.31)	18.2 (0.35)	18.7 (2.48)
Low-average	21.2 (0.41)	16.7 (2.24)	24.8 (0.38)	23.9 (2.68)
High-average	39.3 (0.48)	31.2 (2.99)	31.6 (0.44)	31.3 (2.87)
High	25 (0.47)	41.1 (3.26)	25.4 (0.44)	26.1 (2.62)
	*X*^2^ = 26.7 *p* ≤ 0.001		*X*^2^ = 3.1 *p* = 0.378	
Marital status				
Currently married	65.1 (0.48)	31.8 (3.08)	61.1 (0.44)	42.4 (3.01)
Previously married	8.4 (0.23)	9 (1.65)	18.3 (0.34)	15.7 (2.01)
Never married	26.5 (0.47)	59.2 (3.07)	20.6 (0.37)	41.9 (3.07)
	*X*^2^ = 71.7 *p* ≤ 0.001		*X*^2^ = 56.6 *p* ≤ 0.001	
Employment status				
Employed	72.9 (0.44)	74.9 (2.84)	53.8 (0.46)	62.6 (3.2)
Student	4.7 (0.24)	3.2 (1.14)	4.4 (0.21)	7.3 (1.94)
Homemaker	1.8 (0.14)	2.4 (0.8)	21.2 (0.39)	15.7 (2.23)
Retired	13.4 (0.3)	12.5 (2.27)	15.2 (0.34)	6.1 (1.39)
Other	7.2 (0.27)	7 (1.38)	5.5 (0.21)	8.2 (1.4)
	*X*^2^ = 12.1 *p* = 0.017		*X*^2^ = 11.4 *p* = 0.022	
Income status^a^				
Low	22.4 (0.43)	18 (2.61)	28.5 (0.47)	29.1 (3)
Low-average	23.5 (0.43)	19 (2.66)	25.9 (0.4)	21.4 (2.68)
High-average	28 (0.45)	24.8 (2.78)	24.6 (0.4)	28.4 (2.85)
High	26.2 (0.48)	38.2 (3.11)	21.1 (0.39)	21 (2.38)
	*X*^2^ = 18.4 *p* ≤ 0.001		*X*^2^ = 4.4 *p* = 0.218	
Age (mean, SD)				
	43.5 (0.17)	41.5 (1.04)	44.8 (0.16)	41.1 (1.07)
	*t* = − 2.3 *p* = 0.022		*t* = − 5.1 *p* ≤ 0.001	

*α* = 0.005; ^a^For Income Status, the sample size was reduced (male: heterosexual = 18,664, GB = 344; female: heterosexual = 25,637, LB = 445)

To reduce participant burden, the CIDI was administered in two parts. All participants completed Part I, assessing core mental disorders. Part II, assessing other disorders and correlates, was administered to all participants with any lifetime Part I diagnosis and a probability subsample of other Part I participants. Part II respondents were weighted by the inverse of their probability of selection. Trained lay interviewers administered surveys face-to-face at all survey sites. Translation, back-translation, harmonization, and quality control procedures were similarly standardized at all participating sites [39]. In accordance with the respective Ethics Review boards, verbal or written informed consent was obtained. The authors assert that all procedures contributing to this work comply with the ethical standards of the relevant national and institutional committees on human experimentation and with the Helsinki Declaration of 1975, as revised in 2008.

### Measures

#### Socio-demographics

The variables assessed were gender, age, marital status (married, never married, or previously married), employment status, current income (categorized into country-specific quartiles of gross income per family member in a household), and highest level of education (categorized into country-specific quartiles).

#### Sexual orientation

Sexual orientation was assessed using a single item (“Which of the following best describes your sexual orientation?”) in the part II sample, with the exception of Argentina and New Zealand, where it was assessed in part I. Participants who identified as something other than the answer options listed (“heterosexual or straight”, “homosexual or gay”, or “bisexual”), who were not sure or did not know, as well as those who refused to answer the question or had a missing value were excluded from the sample; females who indicated 'homosexual' will be referred to as ‘lesbian’. In all countries except Argentina, Australia, and New Zealand, participants who reported that they had never had sexual intercourse were not presented with the sexual orientation question, unless they indicated that they had biological children.

#### Mental disorders

Analyses were conducted with 12-month CIDI DSM-IV diagnoses. We included the following mental disorders: mood disorders (major depressive disorder [MDD], dysthymia, bipolar disorder I/II/subthreshold), anxiety disorders (panic disorder, agoraphobia, social phobia, specific phobia, generalized anxiety disorder [GAD], post-traumatic stress disorder [PTSD]), behavioral disorders (attention-deficit/hyperactivity disorder [ADHD], ODD/CD (oppositional defiant disorder and conduct disorder, combined into a single variable due to low prevalence), intermittent explosive disorder [IED]), substance use disorders (alcohol and drug abuse and dependence), and eating disorders (bulimia nervosa and binge eating disorder, combined into a single variable).

#### Social support quality

Social support quality was separately assessed for family and friends using a two-item measure. Participants reported both the frequency of contact (“How often do you talk on the phone or get together with [relatives/friends]?”; “less than once a month”, “about once a month”, “a few times a month”, “a few times a week”, or “most every day”) as well as their general openness to talk to either family or friends about their worries (“How much can you open up to your [relatives/friends] if you need to talk about your worries?”; “not at all”, “a little”, “some”, or “a lot”,). Participants who answered “don’t know” or refused to answer any of the questions, as well as participants with missing values were removed from the sample for the moderation analysis. Social support quality was assessed in a subset of eleven countries (Argentina, Australia, Colombia, Colombia (Medellin), Japan, Mexico, Northern Ireland, Peru, Romania, Spain (Murcia), United States). Notably, the openness item assessed openness with relatives or friends in general, and not specifically related to sexual minority status as the aim of this paper was to assess differences between heterosexual and LGB individuals.

#### Social acceptance

Data on social acceptance were taken from the LGBT Global Acceptance Index [40] which summarizes societal acceptance based on surveys across 141 different countries from 1981 until 2014. For each of the 13 countries included in this study, the value that most closely corresponded to the year that the survey was conducted was selected.

### Analyses

We first examined the prevalence of sexual orientation sub-group to provide readers with an overview of the specific characteristics of the sample as it related to the current study. To this end, we also tested how sociodemographic variables were associated with sexual orientation using linear regression for continuous variables and multinomial logistic regression for categorical variables.

#### First aim

We used logistic regression to test the association between sexual orientation and 12-month mental disorders separately by gender. The decision to conduct gender-stratified analyses was determined a priori based on a review of the literature, which has suggested important gender differences in the associations between sexual orientation and mental disorders [2, 3, 5]. In addition, we used logistic regression to test the association between sexual orientation and 12-month mental disorders comparing LG participants and bisexual participants to heterosexual participants, as well as bisexual participants to LG participants, while controlling for gender. The size of sexual orientation sub-groups did not allow us to conduct this comparison as a gender-stratified analysis. Although our main analyses were completed in the full sample of countries, we additionally performed logistic regressions to test the association between sexual orientation and having at least one 12-month mental disorder separately for each country.

#### Second aim

To assess possible mediation of the association between sexual orientation and psychiatric morbidity by social support, we employed a hierarchical regression approach [28]. First, we repeated a logistic regression analysis with sexual orientation as a predictor and mental disorder as an outcome in the subsample with information on all variables involved in the possible mediation (i.e. sexual orientation, mental disorder, and social support; Model 1). Second, we performed four ordinal logistic regressions with sexual orientation as a predictor and each of the four indicators of social support (contact frequency and general openness with family and friends) as an outcome (Model-set 2). Third, we performed a logistic regression analysis with sexual orientation and all four indicators of social support as predictors and mental disorder as an outcome (Model 3). All models were performed separately by gender. From these three (sets of) models, we calculated the indirect effect of each social support indicator and the total indirect effect by summing the four indirect effects. Estimates of the statistical significance of indirect effects were estimated in 10,000 bootstrap samples using Rao, Wu, and Yue’s bootstrap weighting method for complex surveys as implemented in SAS [41]. Due to concerns about model convergence problems in at least some of the bootstrap samples (which were smaller than the original sample) for uncommon disorders or disorder groups, we examined possible mediation only for any mental disorder and for > 1 mental disorder.

All analyses controlled for the country of origin of the participant. Because the data were clustered and weighted, standard errors were estimated using the design-based Taylor series method implemented in SAS [42]. Statistical significance for all analyses was evaluated at an adjusted *α* of 0.005, to account for the large number of tests employed in the analysis. All analyses were conducted in SAS 9.4.

## Results

### Sample descriptives

Of the initial 48,250 participants (female = 27,882, male = 20,368), 1361 were omitted from the sample, due to reporting their sexual orientation as “something else” (*N* = 180), because they were not sure about their sexual orientation or did not provide a response (*N* = 345), or because they indicated never having had intercourse (*N* = 836, see Supplemental Table 2).

The full sample demographics can be found in Table 1. Out of 46,889 participants (male = 42.4%, female = 57.6%), 0.8% reported being gay/bisexual (*N* = 357) and 1.0% reported being lesbian/bisexual (*N* = 450). More women identified as bisexual (women = 0.9%, men = 0.4%), while more men identified as exclusively same-sex attracted (women = 0.7%, men = 1.0%; see Supplemental Table 3). LGB participants were younger and were less frequently married (currently or in the past) compared to heterosexual participants. There were no differences in employment status among LGB and heterosexual participants; however, GB but not LB participants reported higher educational and income levels than heterosexual participants.

### Sexual orientation and mental disorders by gender

#### Women

Compared to heterosexual women, LB women were significantly more likely to report at least one 12-month disorder (OR 2.2, *p* < 0.001), as well as more than one 12-month disorder (OR 3.3, *p* < 0.001). Among LB women, we found increased prevalence rates for all disorder groups (OR 2.2–4.9, *p* ≤ 0.001). LB women were also more likely to report each specific disorder (OR 2.2–9.4, *p* < 0.001), except for specific phobia (OR 1.7, *p* = 0.007) and ADD (OR 2.0, *p* = 0.116; see Table 2).

**Table 2:** Prevalence of 12-month mental disorders among participants with heterosexual or lesbian/gay and bisexual attraction in all countries

Disorder	Female						Male					
	Heterosexual		Lesbian/bisexual		OR (95% CI)	*p* value	Heterosexual		Gay/bisexual		OR (95% CI)	*p* value
	*N*	% (SE)	*N*	% (SE)			*N*	% (SE)	*N*	% (SE)		
Major depressive episode (non-hierarchical)	2640	7.3 (0.2)	87	15.9 (2)	2.5* (1.9–3.4)	< 0.001	1091	4.2 (0.2)	49	11.3 (1.9)	2.8* (1.9–4.1)	< 0.001
Dysthymia (non-hierarchical)	707	1.9 (0.1)	27	4.6 (1)	2.5* (1.5–4.0)	< 0.001	282	1.2 (0.1)	13	3.0 (0.9)	2.4 (1.3–4.7)	0.008
Bipolar disorder (broad)	642	1.8 (0.1)	33	6.7 (1.5)	4.0* (2.4–6.6)	< 0.001	433	1.7 (0.1)	23	4.9 (1.2)	2.7* (1.6–4.5)	< 0.001
Any mood disorder	2973	8.2 (0.2)	100	19.3 (2.3)	2.8* (2.1–3.7)	< 0.001	1348	5.2 (0.2)	60	14 (2.0)	2.8* (2.0–3.9)	< 0.001
Agoraphobia (with or without panic)	483	1.2 (0.1)	19	3.3 (0.9)	3.1* (1.7–5.3)	< 0.001	171	0.7 (0.1)	10	2.0 (0.8)	2.7* (1.2–6.1)	0.02
Generalized anxiety disorder	1196	3.4 (0.1)	42	7.4 (1.6)	2.2* (1.4–3.4)	< 0.001	479	1.9 (0.1)	19	3.8 (0.9)	1.8 (1.1–3.1)	0.027
Panic disorder (with or without agoraphobia)	625	1.7 (0.1)	30	5.6 (1.4)	3.1* (1.8–5.4)	< 0.001	253	1.0 (0.1)	15	3.5 (0.9)	3.1* (1.7–5.6)	< 0.001
Post-traumatic stress disorder	1069	3.4 (0.1)	55	11.2 (1.7)	3.2* (2.3–4.5)	< 0.001	298	1.4 (0.1)	16	4.6 (1.2)	3.1* (1.7–5.6)	< 0.001
Specific phobia	2769	9.4 (0.2)	63	14 (2.2)	1.7 (1.2–2.4)	0.007	896	4.1 (0.2)	33	9.7 (2.1)	2.3* (1.4–3.8)	< 0.001
Social phobia	1521	4.4 (0.1)	52	11.1 (2)	2.6* (1.7–3.9)	< 0.001	802	3.1 (0.1)	39	11.7 (2.1)	3.7* (2.4–5.7)	< 0.001
Any anxiety disorder	5863	16.9 (0.3)	166	29.7 (2.6)	2.2* (1.7–2.8)	< 0.001	2498	9.7 (0.3)	103	25.3 (2.6)	3.0* (2.2–4.1)	< 0.001
Any eating disorder	268	1.4 (0.1)	13	7.8 (2.5)	4.9* (2.4–9.9)	< 0.001	88	0.6 (0.1)	6	2.1 (1.0)	3.2 (1.1–8.9)	0.028
Attention deficit disorder	143	0.9 (0.1)	6	4.7 (2)	2.0 (0.8–4.7)	0.116	124	1.2 (0.1)	4	2.3 (1.2)	1.2 (0.4–3.4)	0.749
Intermittent explosive disorder	337	1.8 (0.1)	12	4.2 (1.1)	2.3* (1.4–3.8)	< 0.001	322	2.6 (0.2)	10	3.7 (1.2)	1.3 (0.6–2.5)	0.506
Oppositional defiant disorder/conduct disorder	70	0.4 (0.1)	2	5.4 (2.2)	6.6* (2.5–17.3)	< 0.001	92	0.9 (0.1)	2	1.1 (0.8)	0.7 (0.1–3.8)	0.699
Any disruptive behavior disorder	445	2.9 (0.2)	18	13.5 (3.2)	2.3* (1.4–3.9)	0.001	410	4.0 (0.3)	11	5.6 (1.8)	1 (0.5–1.9)	0.927
Alcohol abuse	377	1.3 (0.1)	19	3.9 (1.2)	2.9* (1.6–5.3)	< 0.001	840	3.8 (0.2)	18	4.3 (1.2)	1.1 (0.6–1.9)	0.827
Alcohol dependence	198	0.6 (0.1)	20	3.9 (1.0)	6.1* (3.4–11.1)	< 0.001	419	1.8 (0.1)	18	3.8 (1.1)	2.0 (1.1–3.6)	0.023
Drug abuse	128	0.4 (0.0)	15	3.1 (0.9)	6.9* (3.7–12.8)	< 0.001	282	1.3 (0.1)	13	3.1 (0.9)	2.1 (1.1–4.0)	0.019
Drug dependence	85	0.3 (0.0)	13	2.5 (0.8)	9.4* (4.8–18.4)	< 0.001	161	0.8 (0.1)	10	2.4 (0.9)	3.0* (1.4–6.5)	0.004
Any substance use disorder	525	1.7 (0.1)	36	6.8 (1.4)	3.7* (2.4–5.8)	< 0.001	1155	5.2 (0.2)	36	8.7 (1.6)	1.6 (1.1–2.4)	0.022
Any disorder	7001	20.4 (0.3)	193	35.1 (2.7)	2.2* (1.8–2.8)	< 0.001	3727	15.3 (0.4)	140	34.2 (3.1)	2.7* (2.0–3.6)	< 0.001
> 1 disorder	3056	8.3 (0.2)	122	22.1 (2.1)	3.3* (2.5–4.2)	< 0.001	1605	6.3 (0.2)	71	16.6 (2.3)	2.6* (1.9–3.7)	< 0.001

*< 0.005

#### Men

Compared to heterosexual men, GB men were more likely to report at least one 12-month disorder (OR 2.7, *p* < 0.001) or more than one 12-month disorder (OR 2.6, *p* < 0.001; see Table 2). Specifically, compared to heterosexual men, GB men were more likely to report mood disorders (OR 2.8, *p* < 0.001) and anxiety disorders (OR 3.0, *p* < 0.001), but they were not significantly more likely to have increased prevalence for any other disorder group (OR 1.0–3.2, *p* = 0.022–0.927; see Table 2). Among GB men, we also found increased prevalence rates for most specific mood and anxiety disorders (OR 2.3–3.7, *p* < 0.001), except for dysthymia, agoraphobia, and GAD (OR 1.8–2.7, *p* ≥ 0.008). We did not find elevated prevalence rates for any of the specific disruptive behavior disorders or substance use disorders (OR 0.07–21, *p* = 0.019–0.749), with the exception of drug dependence (OR 3.0, *p* = 0.004).

### Prevalence of mental disorders by sexual orientation

Compared to heterosexual participants, both LG and bisexual participants were significantly more likely to report at least one 12-month disorder as well as more than one 12-month disorder (OR 2.1–2.5, *p* < 0.001; see Table 3). Specifically, both LG and bisexual participants were more likely to report mood disorders, anxiety disorders, and eating disorders than heterosexual participants (OR 2.0–5.3, *p* ≤ 0.004). Bisexual, but not LG, participants additionally were more likely to report substance use disorders (OR 3.7, *p* < 0.001). There were no differences for 12-month disruptive behavior disorders. While bisexual participants, as compared to LG participants, showed somewhat elevated risks for reporting each of the disorder groups, as well as for reporting at least one or more than one disorder, these differences were not significant (OR 1.3–2.2, *p* = 0.009–0.535).

**Table 3:** Prevalence of 12-month mental disorders among participants with heterosexual or lesbian/gay or bisexual attraction in all countries

Disorder	Heterosexual		Lesbian/gay		Bisexual		LG (ref heterosexual)			Bisexual (ref heterosexual)			Bisexual (ref LG)		
	*N*	% (SE)	*N*	% (SE)	*N*	% (SE)	OR	95% CI	*p* value	OR	95% CI	*p* value	OR	95% CI	*p* value
Any mood disorder	4321	6.8 (0.2)	79	15.4 (2.0)	81	18.7 (2.5)	2.4*	(1.8–3.3)	< 0.001	3.2*	(2.4–4.4)	< 0.001	1.3	(0.8–2.1)	0.213
Any anxiety disorder	8361	13.4 (0.2)	127	24.3 (2.2)	142	32.3 (3.2)	2.0*	(1.6–2.6)	< 0.001	3.3*	(2.4–4.4)	< 0.001	1.6	(1.1–2.4)	0.018
Any eating disorder	356	1.0 (0.1)	8	4.4 (1.9)	11	6.7 (2.3)	3.7*	(1.5–9.0)	0.004	5.3*	(2.5–11.1)	< 0.001	1.4	(0.5–4.4)	0.525
Any disruptive behavior disorder	855	3.5 (0.2)	14	7.3 (1.7)	15	11.8 (3.2)	1.3	(0.8–2.2)	0.324	2	(1.1–3.4)	0.015	1.5	(0.7–3.2)	0.291
Any substance use disorder	1680	3.4 (0.1)	34	6.8 (1.3)	38	8.8 (1.7)	1.7	(1.1–2.5)	0.023	3.7*	(2.4–5.6)	< 0.001	2.2	(1.2–4.1)	0.009
Any disorder	10,728	18.0 (0.3)	172	33.0 (2.5)	161	36.9 (3.3)	2.1*	(1.7–2.7)	< 0.001	2.9*	(2.2–3.9)	< 0.001	1.4	(1.0–2.0)	0.08
> 1 disorder	4661	7.3 (0.2)	94	18.4 (2.1)	99	21.2 (2.6)	2.5*	(1.9–3.4)	< 0.001	3.7*	(2.8–5.0)	< 0.001	1.5	(1.0–2.2)	0.077

### Country-level social acceptance and mental health

**Fig. 1:**
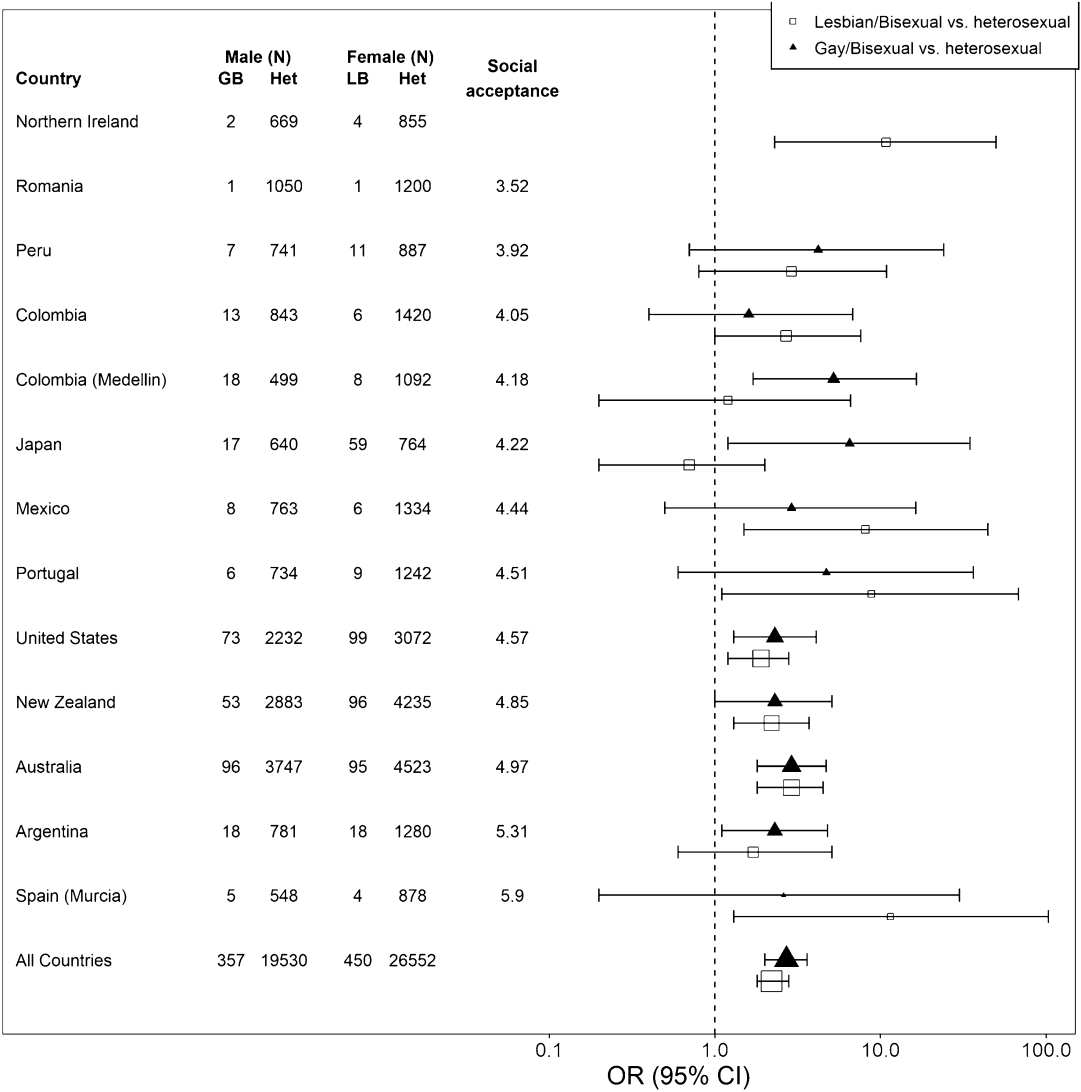
Association between likelidhood of reporting at least one 12-month disorder and country-level social acceptance, by sexual orientation. The social acceptance score for Northern Ireland was omitted, because the index only included an estimate for Great Britain as a whole and Northern Ireland was not included in this estimate. There were zero cases with a 12﻿-month mental disorder among the (very small) sample for GB participants in Northern Ireland and both GB and LB participants in Romania

Figure 1 shows the risk for reporting at least one 12-month disorder among GB or LB participants as compared to heterosexual participants by country. Countries are ranked by their increasing country-level social acceptance scores, illustrating that the relative risk for LB and GB participants to report at least one 12-month disorder did not appear to be associated with country-level LGB social acceptance.

### Mediation by social support

The full results of the mediation analysis in women and men are provided in Supplemental Tables 4–7. The results for Model 1 (sexual orientation predicting mental disorder) were very similar to those discussed before and hence are not discussed here.

#### Women

With regard to Model-set 2 (the association between sexual orientation and social support), we found that LB women were significantly less likely to report high levels of contact frequency (OR 0.6, *p* = 0.004) and high general openness with their family (OR 0.5, *p* < 0.001) than heterosexual women (see Table 4). However, there were no differences between LB and heterosexual women in frequency of contact and general openness with friends (OR 1.1, *p* = 0.720, and OR 0.8, *p* = 0.120, respectively).

**Table 4:** Differences in the distribution of scores on openness and contact frequency with family and friends by sexual orientation, separately by gender

	Males				Females			
	Family		Friends		Family		Friends	
	Heterosexual	GB	Heterosexual	GB	Heterosexual	LB	Heterosexual	LB
	% (SE)	% (SE)	% (SE)	% (SE)	% (SE)	% (SE)	% (SE)	% (SE)
Openness (*N*)	9302	166	9298	166	13,258	204	13,218	206
Not at all	16.71 (0.55)	21.33 (4.59)	19.68 (0.6)	11.82 (3.23)	13.92 (0.49)	22.31 (4.08)	20 (0.54)	11.51 (3.03)
A little	17.98 (0.52)	17.82 (3.09)	19.98 (0.6)	11.45 (3.04)	13.56 (0.43)	17.84 (4.17)	16.21 (0.45)	28.71 (4.95)
Some	26.46 (0.57)	16.25 (3.64)	29.27 (0.64)	27.01 (4.3)	24.51 (0.61)	25.81 (3.2)	25.69 (0.52)	26.90 (3.87)
A lot	38.86 (0.74)	44.60 (4.96)	31.08 (0.73)	49.71 (5.56)	48.01 (0.67)	34.04 (4.1)	38.10 (0.7)	32.88 (4.27)
	OR 0.9, 95% CI 0.6–1.4, *p* value = 0.771		OR 2.0*, 95% CI 1.3–3.1, *p* value ≤ 0.001		OR 0.5*, 95% CI 0.4–0.7, *p* value ≤ 0.001		OR 0.8, 95% CI 0.6–1.1, *p* value = 0.120	
Contact frequency (*N*)	9307	167	9312	167	13,245	206	13,197	206
Most every day	19.47 (0.58)	20.57 (4.03)	21.14 (0.54)	28.36 (4.68)	30.05 (0.72)	19.67 (2.86)	20.02 (0.51)	19.38 (4.13)
Few times a week	29.73 (0.64)	33.23 (5.35)	31.12 (0.68)	34.48 (5.35)	29.29 (0.62)	20.77 (3.7)	27.80 (0.56)	30.51 (4.11)
Few times a month	20.26 (0.53)	17.26 (3.65)	19.43 (0.58)	13.70 (3.04)	16.67 (0.45)	25.07 (4.17)	19.80 (0.49)	23.40 (3.69)
Once a month	10.54 (0.42)	11.43 (2.99)	9.98 (0.39)	9.98 (2.42)	8.83 (0.34)	11.73 (2.7)	9.30 (0.33)	12.03 (2.91)
Less than once a month	20 (0.57)	17.51 (3.86)	18.32 (0.58)	13.48 (3.13)	15.16 (0.47)	22.76 (4.12)	23.08 (0.58)	14.68 (2.61)
	OR 1.2, 95% CI 0.9–1.8, *p* value = 0.248		OR 1.5, 95% CI 1.0–2.2, *p* value = 0.026		OR 0.6*, 95% CI 0.5–0.9 *p* value = 0.004		OR 1.1, 95% CI 0.8–1.4, *p* value = 0.720	

In Model 3, including sexual orientation and the social support indicators as predictors, lower openness with family was significantly associated with having at least one (OR 0.8, *p* < 0.001) or more than one 12-month disorder (OR 0.8, *p* < 0.001). Additionally, a lower frequency of contact with friends was significantly associated with having more than one 12-month disorder (OR 0.9, *p* < 0.001). For at least one 12-month mental disorder, there was a significant indirect effect of openness with family (*ab* estimate 0.129, 99.5% bootstrap percentile interval 0.036–0.250) and a significant total indirect effect of all social support indicators (total *ab* estimate 0.141, 99.5% bootstrap percentile interval 0.036–0.266, proportion mediated 19.0%). Results were similar for more than one 12-month disorder: there was a significant indirect effect of openness with family (*ab* estimate 0.172, 99.5% bootstrap percentile interval 0.047–0.330) and a significant total indirect effect of all social support indicators (total *ab* estimate 0.205, 99.5% bootstrap percentile interval 0.053–0.368, proportion mediated 16.9%).

#### Men

In model 2, GB men reported significantly higher general openness with friends than heterosexual men (OR 2.0, *p* < 0.001; see Table 4). However, there were no significant differences between GB men and heterosexual men in general openness with family (OR 0.9, *p* = 0.771), or frequency of contact with either family or friends (OR 1.2, *p* = 0.248, and OR 1.5, *p* = 0.026, respectively). In Model 3, including sexual orientation and the social support indicators as predictors, lower openness with family was significantly associated with having at least one (OR 0.8, *p* < 0.001) or more than one 12-month disorder (OR 0.8, *p* < 0.001). Additionally, lower frequency of contact with family (OR 0.9, *p* = 0.002) and higher frequency of contact with friends (OR 1.1, *p* = 0.003) were also significantly associated with having at least one mental disorder. However, there were no significant indirect effects, indicating no evidence for mediation.

## Discussion

The findings from this large general population-based study provide cross-national evidence for the disparities in psychiatric morbidity between LGB and heterosexual individuals. Our results are consistent with those reported in national population-based studies [4, 5, 7, 11] and cross-national meta-analyses [20]. We extended these findings by illustrating that this increased risk is a cross-national issue that is present across a range of disorders (particularly for LB women), and further provided additional evidence partially supporting the psychological mediation framework [27].

### Cross-national disparities in psychiatric morbidity

In line with our first aim, our findings add to previous studies documenting the higher likelihood for both LGB women and men to report mood and anxiety disorders compared to heterosexual participants [5, 7, 10]. Furthermore, we found that LB women but not GB men were more likely to report a 12-month substance use disorder compared to heterosexual participants, consistent with previous national studies [2, 3]. The exception to this finding was drug dependence, which was more likely among both sexual minority women and men compared to heterosexual participants. We also found that LB women, but not GB men, showed clearly increased risks for disruptive behavior disorders. Overall, we found a heightened risk for LGB individuals of any gender to report at least one disorder in the past year [5]. In contrast to previous, smaller national studies that found either no difference [5] or elevated risks for comorbidity only among GB men [4]; we found that both GB men and LB women were more likely to report more than one 12-month disorder compared to heterosexual participants. Contrasting previous studies [9], we did not find significant differences in the risk for psychiatric morbidity between LG and bisexual participants, despite the fact that bisexual participants showed elevated rates of psychiatric morbidity compared to LG participants. One possible explanation for this might have been the small size of sexual orientation sub-groups.

While only exploratory and limited by the relatively small number of surveys included, our exploratory analysis suggests no clear relationship between country-level social acceptance and risk for psychiatric morbidity. Instead, an individual’s sexual minority status appears to be a stable risk factor for psychiatric morbidity across countries. This finding is counter to both our expectations and to the literature suggesting a relationship between state-level LGB climate and improved mental health [19, 28]. However, increasing social acceptance of sexual minorities might result in LGB-specific discrimination becoming more subtle rather than disappearing, as Sandfort and colleagues have suggested [5]. To address this, future cross-national research on the relationship between sexual minority stress and psychiatric morbidity should assess both country-level acceptance as well as person-level experiences of discrimination.

### Influence of social support quality

Regarding our second aim, the exploratory mediation analysis found that, among LB women, lower levels of social support partially accounted for the relationship between sexual minority status and the heightened risk for reporting at least one or more than one 12-month disorder. This effect was primarily related to lower openness with family, and we found no evidence for a similar effect among men. These findings are partially consistent with the idea that sexual orientation-based stigmatization may lead to lower social support quality, making sexual minority individuals less resilient to life stressors [27, 28, 32, 34, 43]. Importantly, differences in the mediating role of social processes among LGB participants are in line with some studies that have shown stronger mediating effects among sexual minority women compared to men [44] but are inconsistent with those finding a mediating effect of social support only among sexual minority men [28]. It is noteworthy that, we found only small and inconsistent differences in the quality of social support between heterosexual and LGB individuals, contrasting previous research [11, 29, 34]. Future research should investigate potential differential mechanisms between sexual minority women and men in the mediating role of social support.

### Strengths and limitations

The current study has multiple strengths. First, to our knowledge, this is the first cross-national study assessing mental health disparities between sexual minority and heterosexual individuals, highlighting that the adverse effect of minority stress experienced by LGB individuals is a global issue. In addition, using the CIDI, a validated and reliable interview, we were able to assess a wide spectrum of disorders. Moreover, participants for our study were sampled from the general population, thus preventing the biased estimates that can arise from sampling from organizations and groups within the LGB community [45].

This study also has several limitations. First, responses were collected between 2001 and 2012, meaning that recent social and legal changes (e.g. marriage equality) are not reflected in the data. However, differences in prevalence rates of mental disorders between LGB and heterosexual participants reported in our study were similar to that of more recent studies [6, 10]. Indeed, Sandfort and colleagues [5] have suggested that increasing social acceptance of sexual minorities might result in LGB-specific discrimination becoming more subtle rather than disappearing, which may explain the persistence of disparities in psychiatric morbidity between LGB and heterosexual individuals despite societal change.

Furthermore, the overall small size of the LGB sample suggests underreporting of sexual minority participants, particularly in low/middle-income countries, where only about 0.5% of the population identified as LGB. This might be related to several methodological characteristics. First, participants might have been less likely to disclose potentially socially undesirable information (e.g. sexual minority status) in an interview compared to computer-assisted administration [46]. Second, in most countries, only participants who reported having had sexual intercourse (or having biological children) were asked about their sexual orientation; sexual minority participants may be less likely to have had sexual intercourse or may not regard same-sex sexual activity as being sexual intercourse. Taken together, this means that self-identified LGB participants may not be fully representative of all LGB people, especially in low/middle-income countries. Notably, it is possible that participants who did not disclose their sexual minority status might systematically differ in their risk of reporting psychiatric morbidity than those who did, leading to a potential underestimation of global mental health disparities. In addition, it is possible that those participants who were able to open up about their sexual minority status also experienced higher levels of general social support. Especially among GB men, this might have obscured a potential mediating effect of social support on mental health outcomes.

We also grouped bisexual and lesbian/gay individuals together for the mediation analyses. This is an issue, as studies have shown that aggregating sexual minority groups can obscure differences in particular sub-groups [47]. Finally, the mediation analysis was based on cross-sectional data, so we have no information about the temporal ordering of events. Although it is reasonable to presume that sexual orientation preceded current social support and 12-month mental health problems, we cannot exclude the possibility that mental disorders actually caused a decrease in social support (among LB women), rather than social support mediating the effect of sexual orientation on mental disorders [44].

## Conclusion

In conclusion, this study provides further evidence for an association between sexual minority status and a wide range of psychiatric disorders from a large cross-national sample. Our results are in line with findings from national studies and stress the increased risk for psychiatric disorders among LGB individuals. We found that the increased risk for a psychiatric disorder in the past year for LGB individuals was partially mediated by perceived openness with family among women, but not men. To fully understand the relationship between minority stress and psychiatric morbidity in sexual minority individuals, future studies should consider gender differences in the influence of social support.

## Supplementary Information

Below is the link to the electronic supplementary material.

Supplementary file1 (DOCX 12 KB)

Supplementary file2 (DOCX 12 KB)

Supplementary file3 (DOCX 9 KB)

Supplementary file4 (DOCX 18 KB)

## Data Availability

Access to the cross-national World Mental Health (WMH) data is governed by the organizations funding and responsible for survey data collection in each country. These organizations made data available to the WMH consortium through restricted data sharing agreements that do not allow us to release the data to third parties. The exception is that the U.S. data are available for secondary analysis via the Inter-University Consortium for Political and Social Research (ICPSR), http://www.icpsr.umich.edu/icpsrweb/ICPSR/series/00527.
