# Supplementary material for: Increased risks for mental disorders among LGB individuals: cross-national evidence from the World Mental Health Surveys
Source: Soc Psychiatry Psychiatr Epidemiol. 2022 Jul 19;57(11):2319–32. doi: 10.1007/s00127-022-02320-z (PMC9636102; doi:10.1007/s00127-022-02320-z)
Supplement: Supplementary file 1 — Supplementary file1 (DOCX 12 KB) [file 127_2022_2320_MOESM1_ESM.docx]

| **Supplemental Table 1.** WMH sample characteristics by World Bank income categories*^a^* | | | | | | | | |
| --- | --- | --- | --- | --- | --- | --- | --- | --- |
|  |  |  |  |  | **Sample size** | | |  |
| **Country by income category** | **Survey*^b^*** | **Sample characteristics*^c^*** | **Field dates** | **Age range** | **Part I** | **Part II** | **Part II and age ≤ 44*^d^*** | **Response rate*^e^*** |
| **I. Low and middle income countries** | | |  |  |  |  |  |  |
| Colombia | NSMH | All urban areas of the country (approximately 73% of the total national population). | 2003 | 18-65 | 4,426 | 2,381 | 1,731 | 87.7 |
| Colombia – Medellin^f^ | MMHHS | Medellin metropolitan area | 2011-12 | 19-65 | 3,261 | 1,673 | -- | 97.2 |
| Mexico | M-NCS | All urban areas of the country (approximately 75% of the total national population). | 2001-2 | 18-65 | 5,782 | 2,362 | 1,736 | 76.6 |
| Peru | EMSMP | Five urban areas of the country (approximately 38% of the total national population). | 2004-5 | 18-65 | 3,930 | 1,801 | 1,287 | 90.2 |
| Romania | RMHS | Nationally representative. | 2005-6 | 18-96 | 2,357 | 2,357 | -- | 70.9 |
| **TOTAL** |  |  |  |  | (60,224) | (31,765) | (7,834) |  |
| **II. High-income countries** | | |  |  |  |  |  |  |
| Argentina | AMHES | Eight largest urban areas of the country (approximately 50% of the total national population) | 2015 | 18-98 | 3,927 | 2,116 | -- | 77.3 |
| Australia*^g^* | NSMHWB | Nationally representative. | 2007 | 18-85 | 8,463 | 8,463 | -- | 60.0 |
| Japan | WMHJ 2002-2006 | Eleven metropolitan areas. | 2002-6 | 20-98 | 4,129 | 1,682 | -- | 55.1 |
| New Zealand*^g^* | NZMHS | Nationally representative. | 2004-5 | 18-98 | 12,790 | 7,312 | -- | 73.3 |
| N. Ireland | NISHS | Nationally representative. | 2005-8 | 18-97 | 4,340 | 1,986 | -- | 68.4 |
| Portugal | NMHS | Nationally representative. | 2008-9 | 18-81 | 3,849 | 2,060 | 1,070 | 57.3 |
| Spain-Murcia | PEGASUS- Murcia | Murcia region. Regionally representative. | 2010-12 | 18-96 | 2,621 | 1,459 | -- | 67.4 |
| United States | NCS-R | Nationally representative. | 2001-3 | 18-99 | 9,282 | 5,692 | 3,197 | 70.9 |
| **TOTAL** |  |  |  |  | (85,766) | (48,425) | (10,706) |  |
| **III. TOTAL** |  |  |  |  | (145,990) | (80,190) | (18,540) |  |
|  | | | | | | | | |

*^a^* The World Bank (2012) Data. Accessed May 12, 2012 at: <http://data.worldbank.org/country>. Some of the WMH countries have moved into new income categories since the surveys were conducted. The income groupings above reflect the status of each country at the time of data collection. The current income category of each country is available at the preceding URL.

*^b^* NSMH (The Colombian National Study of Mental Health); MMHHS (Medellín Mental Health Household Study); M-NCS (The Mexico National Comorbidity Survey); EMSMP (La Encuesta Mundial de Salud Mental en el Peru); RMHS (Romania Mental Health Survey); AMHES (Argentina Mental Health Epidemiologic Survey); NSMHWB (National Survey of Mental Health and Wellbeing); WMHJ2002-2006 (World Mental Health Japan Survey); NZMHS (New Zealand Mental Health Survey); NISHS (Northern Ireland Study of Health and Stress); NMHS (Portugal National Mental Health Survey); PEGASUS-Murcia (Psychiatric Enquiry to General Population in Southeast Spain-Murcia); NCS-R (The US National Comorbidity Survey Replication).

*^c^* Most WMH surveys are based on stratified multistage clustered area probability household samples in which samples of areas equivalent to counties or municipalities in the US were selected in the first stage followed by one or more subsequent stages of geographic sampling (e.g., towns within counties, blocks within towns, households within blocks) to arrive at a sample of households, in each of which a listing of household members was created and one or two people were selected from this listing to be interviewed. No substitution was allowed when the originally sampled household resident could not be interviewed. These household samples were selected from Census area data in all countries. Several WMH surveys (e.g. Spain-Murcia) used municipal, country resident or universal health-care registries to select respondents without listing households. The Japanese sample is the only totally un-clustered sample, with households randomly selected in each of the 11 metropolitan areas and one random respondent selected in each sample household.

*^d^* Argentina, Australia, Colombia-Medellin, Japan, New Zealand, Northern Ireland, Romania, and Spain-Murcia did not have an age restricted Part 2 sample. All other surveys were age restricted to ≤ 44.

*^e^* The response rate is calculated as the ratio of the number of households in which an interview was completed to the number of households originally sampled, excluding from the denominator households known not to be eligible either because of being vacant at the time of initial contact or because the residents were unable to speak the designated languages of the survey.

*^f^* Colombia moved from the "lower and lower-middle income" to the "upper-middle income" category between 2003 (when the Colombian National Study of Mental Health was conducted) and 2010 (when the Medellin Mental Health Household Study was conducted), hence Colombia's appearance in both income categories. For more information, please see footnote *a*.

*^g^* For the purposes of cross-national comparisons we limit the sample to those 18+.
