# Supplementary material for: Increased risks for mental disorders among LGB individuals: cross-national evidence from the World Mental Health Surveys
Source: Soc Psychiatry Psychiatr Epidemiol. 2022 Jul 19;57(11):2319–32. doi: 10.1007/s00127-022-02320-z (PMC9636102; doi:10.1007/s00127-022-02320-z)
Supplement: Supplementary file 2 — Supplementary file2 (DOCX 12 KB) [file 127_2022_2320_MOESM2_ESM.docx]

| **Supplemental Table 2. Percentage of participants with a specific sexual orientation, by country and gender** | | | | | | | | | | | | | | |
| --- | --- | --- | --- | --- | --- | --- | --- | --- | --- | --- | --- | --- | --- | --- |
|  | **Women** | | | | | | | **Men** | | | | | | |
| **Country** | **N** | **Heterosexual** | **Lesbian/Gay** | **Bisexual** | **Something**  **else** | **Not**  **sure/missing** | **Skipped**  **out** | **N** | **Heterosexual** | **Lesbian/Gay** | **Bisexual** | **Something**  **else** | **Not**  **sure/missing** | **Skipped**  **out** |
| Colombia | 1496 | 1420 | 1 | 5 | 4 | 5 | 61 | 885 | 843 | 7 | 6 | 5 | 2 | 22 |
| Colombia  (Medellin) | 1142 | 1092 | 2 | 6 | 7 | 4 | 31 | 531 | 499 | 11 | 7 | 3 | 1 | 10 |
| Mexico | 1509 | 1334 | 3 | 3 | 8 | 3 | 158 | 853 | 763 | 6 | 2 | 2 | 1 | 79 |
| Peru | 1004 | 887 | 3 | 8 | 1 | 9 | 96 | 797 | 741 | 2 | 5 | 4 | 7 | 38 |
| Romania | 1265 | 1200 | - | 1 | 32 | 3 | 29 | 1092 | 1050 | - | 1 | 27 | 5 | 9 |
| **Low/middle Income** | **6416** | **5933** | **9** | **23** | **52** | **24** | **375** | **4158** | **3896** | **26** | **21** | **41** | **16** | **158** |
| Argentina | 2235 | 2195 | 15 | 8 | 2 | 15 | - | 1692 | 1647 | 26 | 5 | 3 | 11 | - |
| Australia | 4620 | 4523 | 54 | 41 | - | 2 | - | 3843 | 3747 | 79 | 17 | - | 0 | - |
| Japan | 948 | 764 | 6 | 53 | 2 | 102 | 21 | 734 | 640 | 3 | 14 | 1 | 61 | 15 |
| Spain  (Murcia) | 898 | 878 | 3 | 1 | 1 | 12 | 3 | 561 | 548 | 2 | 3 | 1 | 5 | 2 |
| New Zealand | 7253 | 7090 | 49 | 76 | 25 | 13 | - | 5537 | 5417 | 57 | 23 | 23 | 17 | - |
| Northern  Ireland | 901 | 855 | 2 | 2 | 1 | 2 | 39 | 702 | 669 | 2 | 0 | 0 | 5 | 26 |
| Portugal | 1301 | 1242 | 2 | 7 | 9 | 20 | 21 | 759 | 734 | 5 | 1 | 5 | 6 | 8 |
| United  States | 3310 | 3072 | 53 | 46 | 10 | 24 | 105 | 2382 | 2232 | 49 | 24 | 4 | 10 | 63 |
| **High income** | **21466** | **20619** | **184** | **234** | **50** | **190** | **189** | **16210** | **15634** | **223** | **87** | **37** | **115** | **114** |
| **All countries** | **27882** | **26552** | **193** | **257** | **102** | **214** | **564** | **20368** | **19530** | **249** | **108** | **78** | **131** | **272** |
