# Supplementary material for: Increased risks for mental disorders among LGB individuals: cross-national evidence from the World Mental Health Surveys
Source: Soc Psychiatry Psychiatr Epidemiol. 2022 Jul 19;57(11):2319–32. doi: 10.1007/s00127-022-02320-z (PMC9636102; doi:10.1007/s00127-022-02320-z)
Supplement: Supplementary file 3 — Supplementary file3 (DOCX 9 KB) [file 127_2022_2320_MOESM3_ESM.docx]

| **Supplements 3. Prevalence of sexual orientation by sex.** | | | | |
| --- | --- | --- | --- | --- |
| **Country income level** | **Sex** |  | **Lesbian/Gay** | **Bisexual** |
|  |  | *N* | *% (SE)* | *% (SE)* |
| **High** | Male | 15944 | 1.1 (0.1) | 0.4 (0.1) |
|  | Female | 21037 | 0.8 (0.1) | 1.0 (0.1) |
|  | All | 36981 | 0.9 (0.1) | 0.7 (0.1) |
| **Low-middle** | Male | 3943 | 0.4 (0.1) | 0.4 (0.1) |
|  | Female | 5965 | 0.1 (0.0) | 0.3 (0.1) |
|  | All | 9908 | 0.2 (0.1) | 0.3 (0.1) |
| **All** | Male | 19887 | 1.0 (0.1) | 0.4 (0.0) |
|  | Female | 27002 | 0.7 (0.1) | 0.9 (0.1) |
|  | All | 46889 | 0.8 (0.1) | 0.6 (0.0) |
