# Supplementary material for: Increased risks for mental disorders among LGB individuals: cross-national evidence from the World Mental Health Surveys
Source: Soc Psychiatry Psychiatr Epidemiol. 2022 Jul 19;57(11):2319–32. doi: 10.1007/s00127-022-02320-z (PMC9636102; doi:10.1007/s00127-022-02320-z)
Supplement: Supplementary file 4 — Supplementary file4 (DOCX 18 KB) [file 127_2022_2320_MOESM4_ESM.docx]

**Note:** We calculated the indirect effect of each social support indicator (by multiplying the log odds ratio of sexual orientation predicting the indicator in Model-set 2 with the log odds ratio of the indicator predicting mental disorder in Model 3, the *ab* path in a mediation model) When applicable, we also calculated the proportion mediated by dividing the total indirect effect by the total indirect effect plus the log odds ratio of sexual orientation predicting mental disorder in Model 3 (*ab / (ab + c’)*). To obtain an estimate of the statistical significance of the indirect effects, we estimated the same models in 10.000 bootstrap samples (using the bootstrap weighting method of Rao, Wu, and Yue for complex surveys as implemented in SAS [41]) and calculated the 1-α bootstrap percentile interval for each individual indirect effect, the total indirect effect, and the proportion mediated.

**Supplemental table 4: Mediation analysis of at least one 12-month mental disorder among women**

|  | **Model** | **Outcome** | **Predictor** | **Estimate** | **OR** | **95% CI** | | **p-value** |
| --- | --- | --- | --- | --- | --- | --- | --- | --- |
| 1 | Model 1 | At least one 12-month mental disorder | Sexual orientation | **0.699** | **2.01*** | **1.47** | **2.76** | **<.001** |
| 2 | Model-set 2 | Family – contact frequency | Sexual orientation | **-0.457** | **0.63*** | **0.46** | **0.87** | **0.005** |
| 3 |  | Family - openness | Sexual orientation | **-0.704** | **0.49*** | **0.35** | **0.70** | **<.001** |
| 4 |  | Friends – contact frequency | Sexual orientation | -0.024 | 0.98 | 0.74 | 1.29 | 0.863 |
| 5 |  | Friends - openness | Sexual orientation | -0.241 | 0.79 | 0.57 | 1.09 | 0.145 |
| 6 | Model 3 | At least one 12-month mental disorder | Sexual orientation | **0.602** | **1.83*** | **1.34** | **2.49** | **<.001** |
| 7 |  |  | Family – contact frequency | -0.026 | 0.98 | 0.93 | 1.02 | 0.243 |
| 8 |  |  | Family - openness | **-0.183** | **0.83*** | **0.79** | **0.88** | **<.001** |
| 9 |  |  | Friends – contact frequency | -0.050 | 0.95 | 0.91 | 1.00 | 0.034 |
| 10 |  |  | Friends - openness | 0.003 | 1.00 | 0.95 | 1.06 | 0.924 |
|  | | | | | | | | |
|  | Indirect effects | | | Estimate |  | 99.5% bootstrap  percentile interval | |  |
| 11 | Family – contact frequency (2 x 7) | | | 0.012 |  | -0.019 | 0.055 |  |
| 12 | Family – openness (3 x 8) | | | **0.129** |  | **0.036** | **0.250** |  |
| 13 | Friends – contact frequency (4 x 9) | | | 0.001 |  | -0.024 | 0.028 |  |
| 14 | Friends – openness (5 x 10) | | | -0.001 |  | -0.028 | 0.030 |  |
| 15 | Total (11 + 12 + 13 + 14) | | | **0.141** |  | **0.036** | **0.266** |  |
| 16 | Proportion mediated (15 / 15 + 6) | | | **0.190** |  | **0.059** | **0.501** |  |

**Supplemental table 5: Mediation analysis of more than one 12-month mental disorder among women**

|  | **Model** | **Outcome** | **Predictor** | **Estimate** | **OR** | **95% CI** | | **p-value** |
| --- | --- | --- | --- | --- | --- | --- | --- | --- |
| 1 | Model 1 | More than one 12-month mental disorder | Sexual orientation | **1.172** | **3.23*** | **2.20** | **4.73** | **<.001** |
| 2 | Model-set 2 | Family – contact frequency | Sexual orientation | **-0.457** | **0.63*** | **0.46** | **0.87** | **0.005** |
| 3 |  | Family - openness | Sexual orientation | **-0.704** | **0.49*** | **0.35** | **0.70** | **<.001** |
| 4 |  | Friends – contact frequency | Sexual orientation | -0.024 | 0.98 | 0.74 | 1.29 | 0.863 |
| 5 |  | Friends - openness | Sexual orientation | -0.241 | 0.79 | 0.57 | 1.09 | 0.145 |
| 6 | Model 3 | More than one 12-month mental disorder | Sexual orientation | **1.014** | **2.76*** | **1.89** | **4.02** | **<.001** |
| 7 |  |  | Family – contact frequency | -0.062 | 0.94 | 0.89 | 0.99 | 0.029 |
| 8 |  |  | Family - openness | **-0.245** | **0.78*** | **0.73** | **0.84** | **<.001** |
| 9 |  |  | Friends – contact frequency | **-0.100** | **0.91*** | **0.85** | **0.96** | **<.001** |
| 10 |  |  | Friends - openness | -0.010 | 0.99 | 0.92 | 1.07 | 0.795 |
|  | | | | | | | | |
|  | Indirect effects | | | Estimate |  | 99.5% bootstrap  percentile interval | |  |
| 11 | Family – contact frequency (2 x 7) | | | 0.028 |  | -0.008 | 0.089 |  |
| 12 | Family – openness (3 x 8) | | | **0.172** |  | **0.047** | **0.330** |  |
| 13 | Friends – contact frequency (4 x 9) | | | 0.002 |  | -0.045 | 0.046 |  |
| 14 | Friends – openness (5 x 10) | | | 0.002 |  | -0.035 | 0.040 |  |
| 15 | Total (11 + 12 + 13 + 14) | | | **0.205** |  | **0.053** | **0.368** |  |
| 16 | Proportion mediated (15 / 15 + 6) | | | **0.169** |  | **0.049** | **0.348** |  |

**Supplemental table 6: Mediation analysis of at least one 12-month mental disorder among men**

|  | **Model** | **Outcome** | **Predictor** | **Estimate** | **OR** | **95% CI** | | **p-value** |
| --- | --- | --- | --- | --- | --- | --- | --- | --- |
| 1 | Model 1 | At least one 12-month mental disorder | Sexual orientation | **0.886** | **2.43*** | **1.67** | **3.53** | **<.001** |
| 2 | Model-set 2 | Family – contact frequency | Sexual orientation | 0.388 | 1.47 | 1.05 | 2.07 | 0.024 |
| 3 |  | Family - openness | Sexual orientation | 0.241 | 1.27 | 0.83 | 1.95 | 0.268 |
| 4 |  | Friends – contact frequency | Sexual orientation | 0.344 | 1.41 | 0.98 | 2.03 | 0.065 |
| 5 |  | Friends - openness | Sexual orientation | **0.761** | **2.14*** | **1.34** | **3.43** | **0.001** |
| 6 | Model 3 | At least one 12-month mental disorder | Sexual orientation | **0.923** | **2.52*** | **1.73** | **3.66** | **<.001** |
| 7 |  |  | Family – contact frequency | **-0.095** | **0.91*** | **0.86** | **0.97** | **0.002** |
| 8 |  |  | Family - openness | **-0.208** | **0.81*** | **0.76** | **0.87** | **<.001** |
| 9 |  |  | Friends – contact frequency | **0.089** | **1.09*** | **1.03** | **1.16** | **0.003** |
| 10 |  |  | Friends - openness | 0.013 | 1.01 | 0.93 | 1.10 | 0.759 |
|  | | | | | | | | |
|  | Indirect effects | | | Estimate |  | 99.5% bootstrap  percentile interval | |  |
| 11 | Family – contact frequency (2 x 7) | | | -0.037 |  | -0.102 | 0.013 |  |
| 12 | Family – openness (3 x 8) | | | -0.050 |  | -0.210 | 0.107 |  |
| 13 | Friends – contact frequency (4 x 9) | | | 0.031 |  | -0.018 | 0.101 |  |
| 14 | Friends – openness (5 x 10) | | | 0.010 |  | -0.092 | 0.123 |  |
| 15 | Total (11 + 12 + 13 + 14) | | | -0.046 |  | -0.223 | 0.139 |  |
| 16 | Proportion mediated (15 / 15 + 6) | | | **-** |  | **-** | **-** |  |

**Supplemental table 7: Mediation analysis of more than one 12-month mental disorder among men**

|  | **Model** | **Outcome** | **Predictor** | **Estimate** | **OR** | **95% CI** | | **p-value** |
| --- | --- | --- | --- | --- | --- | --- | --- | --- |
| 1 | Model 1 | More than one 12-month mental disorder | Sexual orientation | **0.737** | **2.09*** | **1.34** | **3.26** | **0.001** |
| 2 | Model-set 2 | Family – contact frequency | Sexual orientation | 0.388 | 1.47 | 1.05 | 2.07 | 0.024 |
| 3 |  | Family - openness | Sexual orientation | 0.241 | 1.27 | 0.83 | 1.95 | 0.268 |
| 4 |  | Friends – contact frequency | Sexual orientation | 0.344 | 1.41 | 0.98 | 2.03 | 0.065 |
| 5 |  | Friends - openness | Sexual orientation | **0.761** | **2.14*** | **1.34** | **3.43** | **0.001** |
| 6 | Model 3 | More than one 12-month mental disorder | Sexual orientation | **0.802** | **2.23** | **1.44*** | **3.46** | **<.001** |
| 7 |  |  | Family – contact frequency | -0.090 | 0.91 | 0.85 | 0.99 | 0.019 |
| 8 |  |  | Family - openness | **-0.195** | **0.82** | **0.75*** | **0.90** | **<.001** |
| 9 |  |  | Friends – contact frequency | 0.084 | 1.09 | 1.01 | 1.18 | 0.036 |
| 10 |  |  | Friends - openness | -0.071 | 0.93 | 0.84 | 1.04 | 0.189 |
|  | | | | | | | | |
|  | **Indirect effects** | | | **Estimate** |  | **99.5% bootstrap**  **percentile interval** | |  |
| 11 | Family – contact frequency (2 x 7) | | | -0.064 |  | -0.197 | 0.056 |  |
| 12 | Family – openness (3 x 8) | | | -0.035 |  | -0.112 | 0.016 |  |
| 13 | Friends – contact frequency (4 x 9) | | | -0.047 |  | -0.205 | 0.097 |  |
| 14 | Friends – openness (5 x 10) | | | 0.029 |  | -0.020 | 0.111 |  |
| 15 | Total (11 + 12 + 13 + 14) | | | -0.054 |  | -0.215 | 0.066 |  |
| 16 | Proportion mediated (15 / 15 + 6) | | | **-** |  | **-** | **-** |  |
